# In vivo gastrin releasing peptide receptor expression in SDH deficient wild-type gastrointestinal stromal tumours (GIST): potential for theranostic applications

**DOI:** 10.1186/s13550-025-01299-3

**Published:** 2025-10-21

**Authors:** David G. E. Hulse, Ines Harper, Fung Tan, Daniel Gillett, Victoria Warnes, Mary McLean, Pascal Wodtke, Ferdia Gallagher, Eamonn R Maher, Olivier Giger, Ramesh Bulusu, Luigi Aloj, Ruth T. Casey

**Affiliations:** 1 Department of Radiology, University of Cambridge, Cambridge, UK; 2 Department of Nuclear Medicine, Cambridge University Hospitals Foundation Trust, Cambridge, UK; 3 Cancer Research UK Cambridge Centre, Cambridge, UK; 4 Department of Medical Genetics, University of Cambridge, Cambridge, UK; 5 Aston Medical School, Aston University, Birmingham, UK; 6 Cellular Pathology Department, St Marks Hospital, London North West University Healthcare NHS Trust, London, UK; 7 Department of Medical Oncology, Cambridge University Hospitals Foundation Trust, Cambridge, UK; 8 Cambridge Endocrine Molecular Imaging Group, Metabolic Research Laboratories, Wellcome-MRC Institute of Metabolic Science, Biomedical Research Centre, University of Cambridge and National Institute for Health Research Cambridge, Addenbrooke’s Hospital, Cambridge Biomedical Campus, Cambridge, UK

## Abstract

**Background:**

Succinate dehydrogenase (SDH) deficient wild-type Gastrointestinal Stromal Tumours (wtGIST) are a rare GIST subtype with limited treatment options. Gallium-68 labelled Gastrin Releasing Peptide Receptor (GRPR) antagonist NeoB has shown promise in PET imaging for multiple primary malignancies. This investigation sought to assess the biodistribution of [^68^Ga]NeoB via PET/CT imaging in metastatic wtGIST patients and aimed to evaluate GRPR expression in lesions to determine the ligand’s potential for patient selection in future therapeutic trials.

**Results:**

Twelve patients with histologically confirmed metastatic wtGIST were enrolled. [^68^Ga]NeoB PET/CT imaging was conducted for lesion segmentation and analysis of uptake characteristics. 8 of 12 (66.7%) patients exhibited intense but heterogeneous [^68^Ga]NeoB uptake in lesions, with variable tracer uptake both within and between lesions. Physiological uptake was highest in the pancreas, liver, and spleen. Four patients (33.3%) displayed minimal or no uptake in tumour lesions.

**Conclusions:**

The majority of wtGIST patients in this small cohort show lesions with intense [^68^Ga]NeoB uptake. Heterogeneity of uptake indicates GRPR has highly variable inter- and intralesional expression. NeoB has potential for theranostic application in wtGIST, with limited effective standard of care treatments available. Ongoing trials are investigating the therapeutic use of [^177^Lu]NeoB in this setting.

**Clinical trial number:**

Not applicable.

**Supplementary Information:**

The online version contains supplementary material available at 10.1186/s13550-025-01299-3.

## Introduction

Wild-type gastrointestinal stromal tumours (wtGIST) are a subset of GIST that lack activating somatic mutations in the *KIT* and *PDGFRA* genes, constituting 15% of adult and 85% of paediatric GIST cases [1]. wtGIST can be further categorized into four molecular subtypes: those with succinate dehydrogenase complex enzyme (SDH) deficiency due to SDH encoding gene (*SDHX*) mutations, those with germline *NF1* mutations, those with somatic *BRAF* mutations, and a fourth group known as ‘quadruple negative’ GIST, lacking mutations in *KIT*, *PDGFRA*, *SDHX*, and *BRAF* genes [2–3].

SDH deficient GIST typically present at a young age, with gastric primary, and exhibit a high rate of metastases [1]. Despite the frequency of metastatic disease at presentation, patients with SDH deficient GIST can still experience survival measured in years, underscoring the necessity for effective treatments with minimal side effects to ensure a high quality of life. Managing wtGIST poses a significant challenge as they respond poorly to standard therapies such as tyrosine kinase inhibitors (TKI). Nonetheless, the primary therapeutic approach for wtGIST currently involves TKIs in the absence of more effective treatment strategies for this population, with notable adverse effects including myopathy and gastrointestinal issues [4]. The lack of promising conventional treatment options for wtGIST has spurred efforts to identify novel molecular targets which could be exploited for therapeutic benefit.

Gastrin-Releasing Peptide (GRP), a bombesin-like peptide growth factor, exerts regulatory control over various physiological processes within the gastrointestinal tract and the central nervous system. These processes include the modulation of gastrointestinal hormone release, smooth muscle cell contraction, and epithelial cell proliferation. The principal mechanism through which GRP exerts its effects is via binding to the Gastrin-Releasing Peptide Receptor (GRPR). Overexpression of GRPR has been reported in multiple cancer types, including GIST, and has been implicated in the promotion of angiogenesis, local invasion, and the formation of distant metastases [5–9]. Given the upregulation of GRPR in neoplasms including GIST there is increasing interest in utilizing this receptor for innovative tumour imaging techniques and targeted cytotoxic therapies. In vitro analysis using three *KIT* mutated GIST derived cell lines demonstrated GRPR expression ranging from 80 to over 95% as assessed using immunohistochemistry (IHC) [10].

NeoBOMB1 (NeoB) is a peptide bombesin analogue acting as an antagonist with high affinity and specificity for GRPR. NeoB contains DOTA metal-chelator, facilitating labelling with radionuclides such as gallium-68 for PET imaging, or lutetium-177 for radionuclide therapy, making NeoB a promising candidate as a theranostic agent [11]. The phase I/II MITIGATE trial evaluated the safety, biodistribution, dosimetry, and initial diagnostic efficacy of [^68^Ga]NeoB in patients with *KIT* or *PDGFRA* mutated GIST. 9 patients with advanced TKI-pre-treated GIST were included, with 6 of 9 patients demonstrating [^68^Ga]NeoB uptake in tumour lesions. Assessment of GRPR expression on biopsy specimens also demonstrated moderate to strong GRPR expression in 6 out of 9 patients, with a correlation between GRPR expression measured by IHC and [^68^Ga]NeoB uptake identified in 3 out of 9 patients [12–13]. NeoB has also been investigated in the context of multiple other primary malignancies. The NeoFIND study assessed the diagnostic efficacy of [^68^Ga]NeoB for identifying GRPR positive tumour lesions in patients with breast, prostate, colorectal, and lung cancers [14–15]. Nineteen patients were enrolled, with [^68^Ga]NeoB identified in at least one lesion in 17 of 19 patients. Among these, all five breast cancer patients exhibited moderate to strong uptake of [^68^Ga]NeoB in the majority of their metastatic lesion, with all samples analysed by immunohistochemistry confirming positive GRPR staining. Alternative tracers targeting GRPR have been investigated. [^68^Ga]-BZH3, demonstrated uptake of 8 out of 30 lesions across 17 patients with GIST [16]. The monoclonal antibody (mAb) RM2 has also been investigated in the context of multiple malignancies including prostate and breast cancer [17–18], however no studies in the context of GIST have been reported.

### Aims

The main objective of this physiological study was to evaluate the bio-distribution of [^68^Ga]-NeoB in patients with metastatic wild-type GIST using PET/CT and to estimate in vivo expression of GRPR in wtGIST tumour(s). A subsequent aim was to facilitate identification of patients who may be eligible for treatment with [^177^Lu]NeoB.

## Methodology

### Ethical approval and study registration

The study was approved by the East of England South Cambridge Research Ethics Committee (REC ID 14/EE/1059). All participants provided written informed consent.

### Clinical data collection

12 patients (9 Female, 3 Male) with histologically confirmed metastatic *SDH* deficient wtGIST were enrolled. Patients were recruited from the National Paediatric and Adult wild-type GIST (PAWS GIST UK), and clinical genetics clinic at Cambridge University Hospital NHS Foundation Trust. Details of clinical phenotype, histopathological subtype, family history, and germline molecular testing results were collated from patient records. Most of these patients were referred to the national clinic from other secondary or tertiary referral centres across the UK and cross-sectional imaging and 18 F FDG PET/CT imaging was performed at local centres and transferred for review for the purpose of this study.

### [^68^Ga]NeoB imaging

The radiopharmaceutical was supplied as a sterile 2-vial kit for the preparation of [^68^Ga]NeoB, allowing for direct ^68^Ga-labeling based on reconstitution of a pre-formulated Good Manufacturing Product kit with the eluate of an approved ^68^Ge/^68^Ga generator. Materials were supplied by Advanced Accelerator Applications (Saint-Genis-Pouilly, France). The volume of [^68^Ga]NeoB solution injected, thus dose of radioactivity administered, was calculated according to the estimated time of injection on the bases of the current activity provided by the generator and the physical decay of the radionuclide (half-life = 67.8 min).

3MBq/Kg (± 10%) of [^68^Ga]NeoB was administered (mean 215.6 MBq, range 156.4–255.9 MBq). Scanning was started after urinary bladder emptying. The mean uptake time was 63.4 min (range 60–72). Image acquisition was performed on a GE Discovery MI PET/CT scanner (GE Healthcare, Milwaukee, WI, USA), (25 cm axial field of view, 4 min per bed position, vertex to upper thighs) with an unenhanced low-dose CT for attenuation correction and localization. Emission data were corrected for decay, dead time and random coincidences and normalized for injected dose and patient body weight. Images were reconstructed using a Bayesian penalized-likelihood algorithm (Q-clear).

All studies were evaluated by experienced nuclear medicine physicians (LA/ IH) for clinical review and governance reporting. Lesions and areas of abnormal uptake were evaluated in each patient. A four-point certainty scoring scale was applied (definitely negative, equivocal probably negative, equivocal probably positive, definitely positive). Lesions with an SUV_max_ uptake ratio < 1 compared with background liver SUV_mean_ were considered to have low/no receptor expression. A ratio of 1–2 was considered equivocal, and a ratio > 2 was defined as representing high receptor expression [19]. Images were analysed for quantitative parameters using 3DSlicer. Maximum standardized uptake values (SUV_max_) were obtained for tumour lesions for each patient and disease site. SUV_mean_ and SUV_max_ were recorded for normal background liver, pancreas, spleen, and the mediastinal blood pool. Uptake attributable to liver metastases, gastric/ peritoneal disease, and other sites of solid organ involvement were segmented manually. The volume of [^68^Ga]NeoB avid disease (defined as SUV > background liver SUV_max_) was derived for each patient and disease site.

### Standard of care imaging

No additional cross-sectional imaging was undertaken for this study. Standard of care staging imaging performed locally or at referring centres was reviewed alongside [^68^Ga]NeoB images for each patient. The most recent correlative contrast enhanced staging CT or MRI study was manually segmented in 3DSlicer, and the volume of gastric/ peritoneal disease, liver metastases, and other sites of solid organ involvement were manually segmented.

6 patients had contrast enhanced CT imaging and 6 patients had contrast enhanced MRI for the most recent comparison study. For 2 patients who had recently (< 3 months) undergone [^18^F]FDG-PET/CT, the volume of FDG avid disease was also derived.

### Statistical analysis

The study of a rare disease makes a power calculation more challenging in addition to a paucity of previous studies, but a sample size of 12 was selected for this pilot study. Statistical analyses were primarily descriptive.

The targeting properties of [^68^Ga]NeoB was assessed for the presence of uptake within known tumour lesions, SUV values per disease site, and tumour-to-background liver ratio of [^68^Ga]NeoB. The presence or absence of [^68^Ga]NeoB uptake was correlated with clinical findings including age at diagnosis, the presence of metastatic disease at diagnosis, histological subtype (epithelioid/ mixed/ spindle cell), *SDHx* mutation (*SDHA* vs. non-*SDHA*), time interval from last reported stable disease to progression, and time interval from last reported disease progression to Neo-B PET. Statistical analysis was performed in Microsoft Excel. Appropriate tests of association were performed where relevant.

**Table 1:** Clinical and molecular features of patient cohort

Case	Age at diagnosis	Sex	Genotype	Primary	Metastatic at presentation	Sites of metastatic disease	Previous therapy	Time between last reported Stable Disease to last reported Disease Progression (months)	Time from last reported Disease Progression to NeoB PET (months)
1	22	M	*SDHB c.72 + 1G > T*	Gastric	Yes	Liver, LN	TKI	Mixed response	Mixed response
2	55	F	*SDHA c.1433-1G > A*	Gastric	Yes	Liver	Partial gastrectomy, TKI, SIRT, EBR	2.5	1.2
3	25	F	*SDHC epimutation*	Gastric	No	Liver	Partial gastrectomy, TKI	4.2	0.8
4	21	M	*SDHA* c.91 C > T (p.Arg31Ter)	Gastric	No	Liver	Partial gastrectomy, TKI, RFA	25.2	4.9
5	31	F	*SDHA* c.1234G > T *(p.Gly412Cys)*	Gastric	Yes	Liver, LN	TKI	2	1.6
6	35	F	SDHA c.1765 C > T (p.Arg589Trp)	Gastric	No	Liver, peritoneal	Partial gastrectomy, TKI	5.3	3.8
7	15	F	SDHA c.91 C > T (p. Arg31Ter)	Gastric	No	Liver, peritoneal, retroperitoneal	Partial gastrectomy, TKI, TAE	5.8	17.4
8	14	F	*SDHA* c.91 C > T, (p.Arg31Ter) & *concurrent SHDC epimutation*	Gastric	Yes	Liver, peritoneal	TKI, debulking	5.2	4.5
9	17	M	*SDHC* del exon 4–6	Gastric	Yes	Liver, peritoneum, seminal vesicles	Debulking Capecitabine/temozolomide	1.8	1.7
10	28	F	*SDHC epimutation*	Gastric	Yes	Liver, LN	Nil	NA	2.5*
11	33	F	Unknown	Gastric	Yes	Liver	Debulking, TK	5.3	4.3
12	26	F	*SDHA* c.1909–2 A > G	Gastric	No	Liver, LN	Gastrectomy, liver resections, TKI, SIRT,	27.9	25.2

(* Patient 10 has no reported progression since diagnosis. Value stated is interval since initial diagnosis)

## Results

### Genotype and clinical phenotype of study cohort

12 patients were included in this study (9 female, 3 male). The median age at presentation was 25.5 years (SD 11.2) and 7 patients had metastatic disease at presentation. All 12 patients had a gastric primary GIST, and the liver was the most common site for metastatic spread (100%).

Six patients had a pathogenic germline variant in *SDHA*, one patient each had a pathogenic *SDHB* and *SHDC* variant respectively. Two patients had negative germline testing but were diagnosed with tumoural hypermethylation of the *SDHC* promoter region (*SDHC* epimutation) and one patient had a co-existing germline *SDHA* variant and an *SDHC* epimutation (Table 1). In one patient, no causative pathogenic variant has been identified however tumour sequencing has confirmed wild type *KIT/ PDGFRA* and *BRAFI* loci, germline genetic testing has not identified an *SDHx* variant but immunohistochemistry has demonstrated SDH deficiency in the tumour. For the 9 patients with accessible histopathology reports on initial biopsy or resection specimen, 4 cases were of epithelioid subtype, 4 of mixed epithelioid/ spindle cell, and 1 of spindle cell subtype. The reported proliferation index was variable, ranging from 0 to 3 mitotic figures per high powered field.

**Fig. 1:**
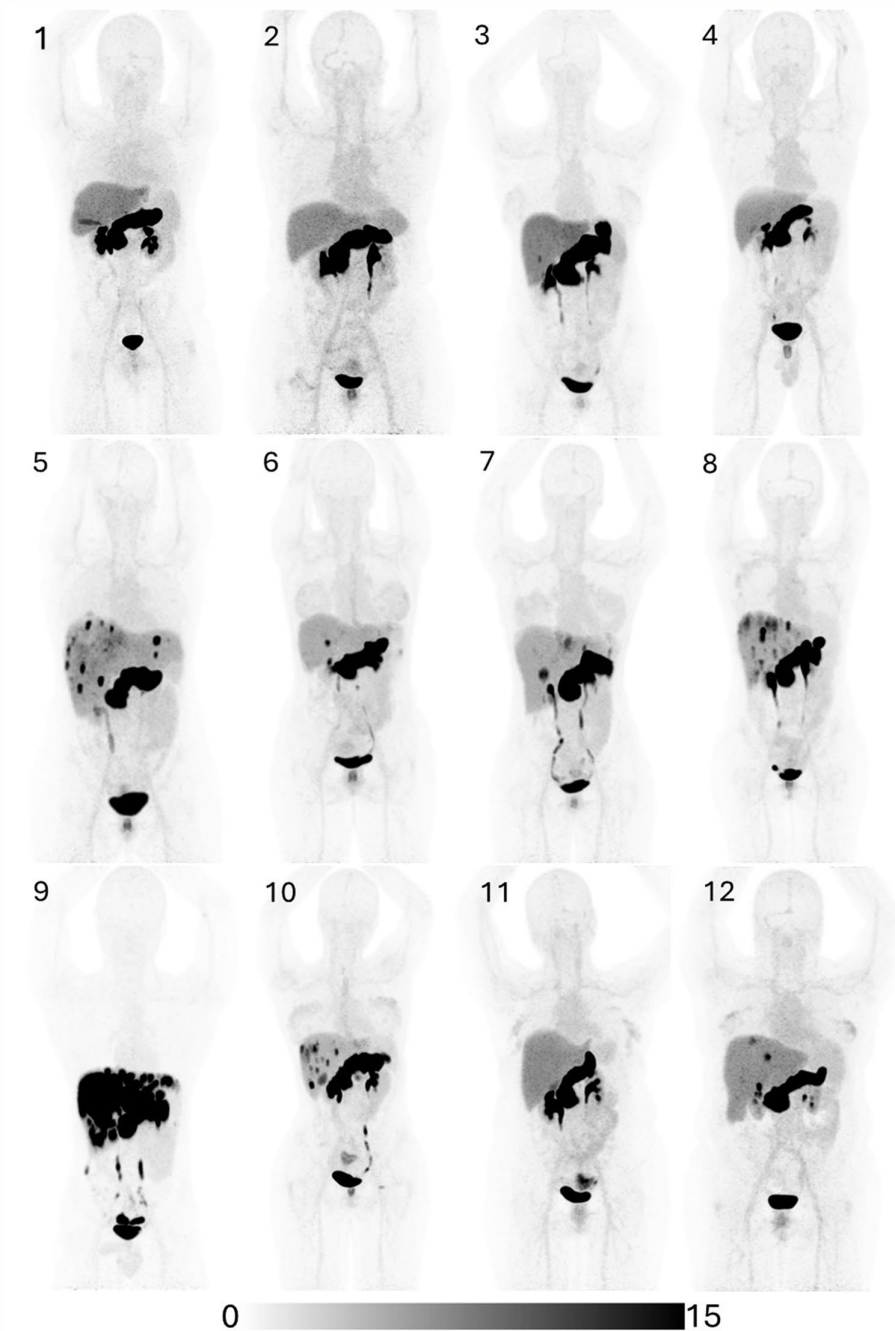
Maximum intensity projection (MIP) images (display threshold SUV 0–15) of [^68^Ga]NeoB uptake in each of the 12 patients enrolled. High physiological uptake within the pancreas in all patients, with variable uptake/ hepatobiliary excretion within the gallbladder. Patients 3, 5, 6, 7, 8, 9, 10, & 12 demonstrate regions of avid uptake within tumour lesions. Patients 1, 2, 4, and 11 demonstrate no or minimal uptake

### Tracer uptake and SUV_max_

[^68^Ga]NeoB PET imaging was obtained for all twelve patients included in this study (Fig. 1). Imaging findings are summarized in Table 2. Physiological uptake was highest in the pancreas, as expected (mean SUV_mean_ 45.4; range 29.3–89.9), followed by the liver (mean SUV_mean_ 4.8; range 3.2–5.8), spleen (mean SUV_mean_ 2.5; range 1.3–4.5), and blood pool (mean SUV_mean_ 2.1; range 0.6–2.9). Focal uptake greater than liver background was also noted within the gallbladder fundus in 5 of 11 patients (1 patient having undergone cholecystectomy), although it is unclear if this was within the gallbladder wall or represented hepatobiliary excretion of the radiopharmaceutical. Additionally, intense uptake was observed in the uterus in one patient (SUV_max_ 17), with no corresponding metastatic disease identifiable on prior or subsequent cross sectional or ultrasound imaging, suggesting potential variability in physiological GRPR expression patterns.

Two patients demonstrated no [^68^Ga]NeoB uptake above liver background in tumour lesions. Two further patients demonstrated tumoural uptake only marginally higher than background liver and in a very small proportion of the disease volume (< 1 ml by volumetric analysis).

The majority of patients (8/12) enrolled in our study exhibited some level of uptake in lesions above background liver. Eight of 12 (66.7%) patients demonstrated intense but heterogeneous uptake in known tumour lesions. For these patients, the mean SUV_max_ for the most avid lesion was 41.3 (mean SUV_max_ 11.2 times greater than background liver SUV_mean_; range 3.9–22.7).

**Fig. 2:**
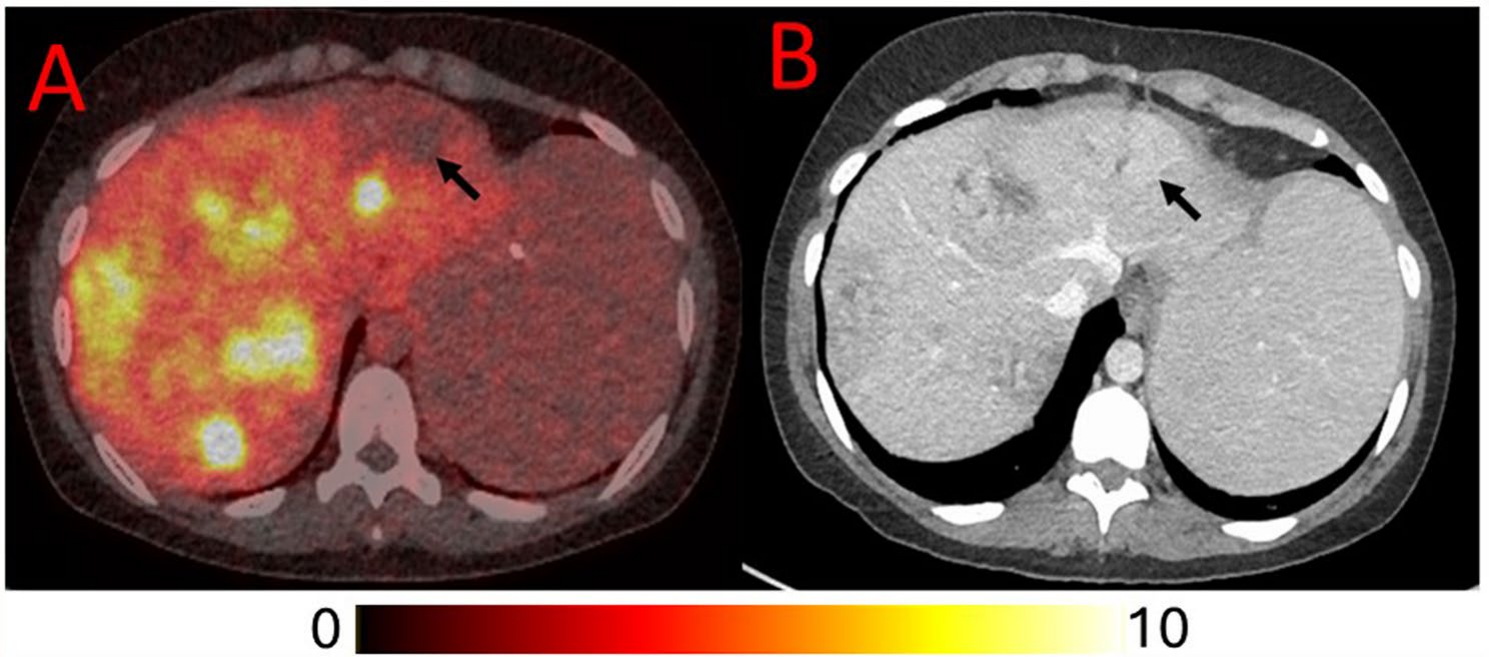
(**A**) Axial fused [^68^Ga]NeoB PET/CT demonstrating heterogenous uptake within liver metastases and (**B**) Correlative staging CT Study showing the extent of liver disease. Multiple enhancing lesions in the right lobe demonstrate NeoB uptake, however a further enhancing lesion within the left lateral section (arrow) corresponds to a region on photopaenia on the PET images

**Fig. 3:**
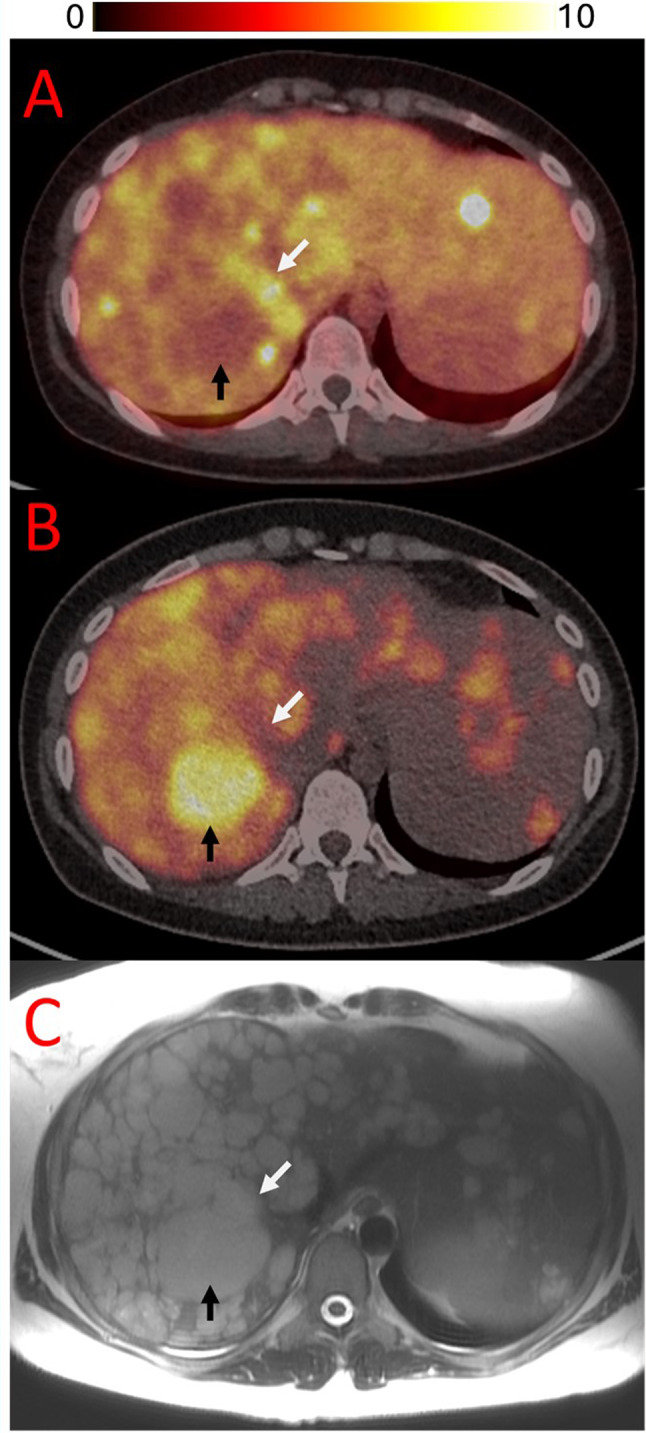
(**A**) Axial fused [^68^Ga] NeoB PET/CT demonstrating heterogenous uptake between liver metastases. (**B**) Axial fused [^18^F]-FDG PET/CT image and (**C**) correlative staging T2w MRI study at the same level. Multiple liver lesions are present, with differential uptake of NeoB and FDG. A lesion indicated in the right lobe demonstrates avid FDG uptake but photopaenia on NeoB PET (black arrow). Conversely, an adjacent lesion (white arrow) demonstrates uptake on NeoB PET without substantial FDG uptake

Substantial heterogeneity in uptake was observed, with lesions demonstrating high avidity mixed with regions with little or no uptake, indicating high variability of the level of GRPR expression both within and between lesions. A proportion of lesions demonstrated uniformly avid uptake of NeoB, however many further lesions exhibited mixed or entirely photopaenic uptake patterns, even in lesions with imaging features of viability, e.g. contrast enhancement or FDG avidity (Figs. 2 and 3). This finding was present both for liver metastases and for gastric/ peritoneal disease. No clear association was noted between tracer uptake and the presence or absence of contrast enhancement within lesions on correlative cross-sectional imaging. In some instances, lesions displayed peripheral uptake with central photopaenia, consistent with areas of necrosis. As expected, there was low or absent uptake within lesions previously managed with local therapy such as selective internal radiotherapy or radiofrequency ablation. NeoB PET imaging identified previously occult metastatic involvement of the seminal vesicles in one patient, which was not recognized on the preceding cross sectional staging imaging (patient 9).

Uptake above background was also noted within a lesion arising from the pelvic musculature in patient 10, previously characterized via MRI as a synovial giant cell tumour, and within a biopsy proven benign fibroadenoma of the breast in patient 5.

**Table 2:** Imaging characteristics of normal tissues and tumour lesions

	Background SUV_mean_						Lesion SUV_max_			
Case	Liver	Spleen	Blood pool	Pancreas	Liver	Gastric/ peritoneal	Other lesion site	Other lesion SUV_max_	Lesion SUV_max_: background SUV_mean_	Governance clinical report
1	5.8	2.8	2.3	89.9	11.2	-	-	-	1.9	Equivocal negative
2	5.5	4.5	2.9	47.1	10	-	-	-	1.8	Equivocal negative
3	7.4	3.4	1.7	48.5	19.7	70	-	-	9.5	Positive
4	4.8	2.5	2.3	39.3	NA	-	-	-	< 1	Negative
5	4.3	2.5	2.2	45.8	53.3	35.3	Spleen	7.3	12.5	Positive
6	4.2	2.2	1.8	43.8	19.8	11.3	Thoracic nodes	6.8	4.8	Positive
7	4.3	1.7	1.9	38.1	16.6	14.3	-	-	3.9	Positive
8	4.1	1.6	2.5	31.8	26.1	30.3	Thoracic nodes	10.5	7.4	Positive
9	3.2	1.3	0.6	29.3	61.6	72.4	Seminal vesicles	48.1	22.7	Positive
10	3.5	1.8	1.5	44.9	42.2	49	-	-	14.1	Positive
11	5.8	2.8	2.5	44.1	NA	-	-	-	< 1	Negative
12	5	2.7	2.8	42.6	19.1	-	-	-	3.8	Positive

[^68^Ga]NeoB imaging findings and SUV_mean_ of normal tissues, SUV_max_ of tumour lesions, and ratio of SUV_max_ from most avid lesion to background liver SUV_mean_

### Volumetry and quantification of avid disease

Volumes of disease burden on cross sectional imaging were manually segmented and compared with the volume of avid disease uptake attributable to metastases in different locations for each patient. These results are summarized in the supplementary Table 1.

Tumour volumes were manually segmented for analysis, permitting the quantitative assessment of disease burden. The volume of disease identified on cross-sectional imaging was greater than identified via [^68^Ga]NeoB PET/CT in 7 of the 8 patients with positive studies (mean percentage of NeoB avid disease = 60% (range 16–180%), using cross sectional imaging as the reference standard). In one patient, the volume of disease identified via NeoB PET was > 100% of the volume identifiable on cross sectional staging MRI; this observation likely results from a combination of rapidly progressive disease and count overspill from areas of intensely avid uptake.

Two patients underwent [^18^F]-FDG PET/CT imaging within 90 days of the NeoB PET study (patients 5 & 10), and in each case the volume of disease identified via FDG was greater than via NeoB (mean NeoB avid = 22.5%; range 16–29%; using FDG as reference standard). This indicates that [^68^Ga]NeoB PET imaging may underestimate the volume of disease, but larger prospective studies are needed to further investigate this finding. Interestingly, in one patient small regions of [^68^Ga]NeoB uptake were present in lesions with low/ no corresponding [^18^F]-FDG uptake (Fig. 3).

### Statistical analysis

Clinical and pathological phenotypes were investigated for predictors of GRPR expression, however no statistically significant correlation was detectable within this sample of 12 patients. Mann-Whitney U test was used to test for association between the presence of NeoB avid disease and continuous variables; age (U = 18, *p* = 0.28), interval from diagnosis to NeoB PET (U = 11, *p* = 0.92), proliferation index (U = 8, *p* = 0.61), time interval from last stable disease (SD)to progressive disease (PD) (U = 10.5, *p* = 0.38), and time interval from last PD to NeoB PET (U = 11 *p* = 0.53). Chi-square test of independence was performed for comparison between the presence of NeoB avid disease and the presence of metastatic disease at presentation (Chi-square 0.0, *p* = 1.00), histological subtype (epithelioid/ mixed/ spindle cell) (Chi-square 0.5, *p* = 0.92), and SDH mutation status (SDHA v. non SDHA pathogenic variant) (Chi-square 0.23, *p* = 0.63).

### Safety profile

Vital signs remained stable throughout the imaging procedure, and there were no significant changes to laboratory studies changes post-imaging. No adverse or clinically detectable pharmacologic effects were observed in any of the 12 study participants during the 12 month follow-up period.

## Discussion

This is the largest study to date investigating in vivo GRPR expression in patients with genetically characterized GIST. Notably, all patients included in this study have SDH deficient wtGIST, a molecular subtype of GIST affecting younger patients and with a high rate of metastatic disease and with limited standard of care treatment options [1]. The availability of effective systemic therapeutic options for patients with metastatic SDH deficient wtGIST represents a significant unmet need in clinical practice. This rare subtype of GIST affects young patients and although metastatic disease is common at presentation [1], the disease can often run an indolent clinical course, highlighting the need for well tolerated but effective treatments for this patient cohort. Eight out of twelve patients (66.7%) demonstrated intense but heterogeneous uptake in known tumour lesions on NeoB PET and would consequently meet imaging criteria for inclusion into the NeoRay study, a phase I/IIa study investigating [^177^Lu]NeoB in patients with advanced solid tumours and with [^68^Ga]NeoB lesion uptake (NCT03872778).

One of the striking features of the PET imaging is the degree of heterogeneity observed in this study. Intratumoural and intertumoural heterogeneity was noted and could not be explained by tumour specific considerations such as necrosis or haemorrhage on correlation with cross sectional imaging (Fig. 3). The mechanisms underpinning this finding remain uncharacterised however it could be speculated that tumour regions demonstrating lower GRPR expression are less well differentiated than those expressing high levels of GRPR. Intratumoural and intertumoural heterogeneity using PET tracers in isolation or combined PET tracers has proven utility in other tumour types for predicting disease progression or tumour de-differentiation [20]– [21]. The limited availability of tissue for analysis in this study prevented further testing of tumour tissue to better understand underlying molecular determinants driving tumour heterogeneity. Previous work undertaken in patients with *KIT* or *PDGFRA* mutant GIST has also demonstrated heterogeneity in NeoB uptake between lesions, suggesting that heterogeneity of GRPR expression may be a feature common to multiple GIST subtypes [13].

Two patients had [^18^F]-FDG PET/CT within a three-month window of their [^68^Ga]NeoB PET study, with NeoB tending to underestimate the volume disease compared with FDG, however with small regions of tumour demonstrating high NeoB uptake without correspondingly elevated FDG uptake. The absence or reduced FDG avidity in some tumour lesions despite high NeoB tracer uptake is of particular interest as patients with *SDHx* variants have increased FDG uptake owing to increased expression of glucose transporters driven by increased anaerobic glycolysis in SDH deficient tumours resulting from a pseudohypoxic metabolic environment [22]. This study was not designed to examine differences in tracer bio distribution but was an interesting observation and the biological mechanisms underpinning the discordance between FDG and NeoB uptake require further investigation in larger prospective studies. Future work investigating GRPR receptor imaging in *SDHx* tumour types should consider employing both tracers and ideally obtain correlative targeted biopsies of discordant regions of tumour uptake. Longitudinal follow-up for lesion-specific progression could also be considered to determine whether the uptake patterns assessed via NeoB/ FDG are associated with a more indolent or aggressive disease course. If an individual is found to have large regions of discordant NeoB/ FDG avid disease, both tracers could be employed in a complimentary manner to assess the overall disease burden.

Previous work undertaken on histology specimens obtained before or after treatment has demonstrated an association between TKI therapy and reduced GRPR expression [23]. Of note, only two patients within this study are TKI naïve (patients 9 &10), and both demonstrate significant NeoB uptake, although patient 10 also demonstrated regions of low uptake suggesting multiple biological factors may be involved with regulating GRPR expression in this cohort. Nonetheless, the histological data suggesting reduced expression with TKI therapy may have implications for the sequencing of treatment with GRPR-targeting Peptide Receptor Radionuclide Therapy (PRRT), but further studies are required to understand the impact of treatment duration with TKI therapy, the effect of stopping therapy on GRPR expression, and the optimum timing between cessation of therapy and NeoB imaging.

NeoB PET underestimated the burden of disease compared with contract enhanced staging MRI or CT imaging for all but one patient, and for both patients for whom recent [^18^F]-FDG PET/CT was available. Nonetheless, NeoB PET identified previously occult seminal vesicle metastases in one patient, suggesting possible diagnostic utility in addition to conventional cross-sectional imaging.

There were no statistically significant correlations identified between in vivo GRPR expression and clinical features or molecular subtypes of SDH deficient wtGIST in this small cohort. As a rare disease, further probing of clinical or molecular predictors of GRPR expression in patients with SDH deficient GIST will likely require a multicentre or internationally coordinated approach.

For the purposes of this study the threshold for defining avid disease on ^68^Ga-NeoB PET imaging was taken to be any uptake within lesions greater than background liver SUV_max_. As a result, there is potential for a small variation in the liver SUV_max_ to have a large impact on the volume of disease classified as avid. Nonetheless, background liver is considered to be the most appropriate reference, as the liver was the most common site of metastasis in this patient cohort and constituted the majority of disease volume for most patients. Therefore, achieving uptake within tumour greater than that of background liver would likely be necessary for targeting GRPR as a therapeutic ligand in this cohort. Alternative background reference standards could be considered in different GRPR expressing malignancies with differing uptake patterns and distributions of metastases.

The derivation of tumour volumes represents another area of complexity; prior cross sectional staging imaging had been performed at multiple different centres and via variable modalities (CT v. MRI), slice thickness, contrast dose, and contrast phase timing. Regions of liver parenchymal abnormality were segmented as disease, however this likely resulted in an overestimation of the true volume of viable liver metastatic disease due to inclusion of regions of necrotic tumour and parenchymal oedema. These factors introduce uncertainty in disease volume estimation and may contribute to inaccuracies in assessing the overall disease burden. As a consequence, the value presented for the overall percentage of NeoB avid disease is likely an underestimate for most patients. Nonetheless, the derived volumes are considered in the opinion of the authors to be a useful means to approximate the disease volume and present a quantifiable method of estimating the extent of NeoB-PET avid disease.

In recent years there has been increasing interest in PRRT, in which a ligand conjugated with an alpha- or beta-emitting radionuclide delivers radiation to a target-expressing tumour. Examples of widely used clinical agents include somatostatin analogues (e.g. Octreotide) and prostate specific membrane antigen (PSMA), typically conjugated with the beta-emitting radionuclide Lutetium-177. An aim of this study was to facilitate identification of patients who may be eligible for [^177^Lu]NeoB therapy as part of a clinical trial or in future clinical practice. 8 of the 12 patients enrolled would be eligible for enrolment in NeoRay on the basis of having at least one measurable lesion per RECIST 1.1 demonstrating regions of [^68^Ga] NeoB uptake on PET/CT. Further, the application of volumetry provides an estimate of the proportion of the overall disease burden in which there is likely to be significant uptake of [^177^Lu]NeoB.

As [^68^Ga]NeoB background uptake is highest in the pancreas by a substantial margin, this is likely to be the organ most at risk of incurring dose limiting toxicity during future therapeutic trials. No consensus radiation dose limit for the pancreas is reported in the literature [24], and although the pancreas is not deemed to be an organ at risk for radiotherapy planning, prior work on dose loading of abdominal organs via External beam radiotherapy (EBR) has shown that grade 1 pancreatic toxicity starts in the region of 60 Gy [25]. While the timecourse for [^68^Ga]NeoB has previously been determined [26], [^177^Lu]-NeoB may exhibit different retention time in pancreas. It is however plausible that similar binding affinity may be achieved. [^177^Lu]-NeoB dosimetry performed in a mouse model did not identify any biochemical or histological pancreatic toxicity, and suggests that dose to kidney could be a limiting factor [27]. Furthermore, while a dosimetry study using the GRPR antagonist [^177^Lu]-RM2 in the context of prostate cancer found initially high pancreatic uptake, rapid release of the compound from the pancreas was observed resulting in an absorbed dose within tolerable limits [28]. The investigators determined that it would be possible to administer a cumulative dose of up to 56 GBq of [^177^Lu]Lu-RM2 over multiple treatment cycles before approaching the 60 Gy threshold. This suggests that GRPR targeting ligands could potentially be employed without incurring pancreatic toxicity in therapeutic trials.

Although the majority of patients in this study had regions of increased tracer uptake within tumour, most demonstrated significant heterogeneity of tumoural GRPR expression. Employing NeoB as a therapeutic agent may leave these regions of tumour untreated and could result in positive selection of clones not expressing GRPR leading to resistance, but we eagerly await treatment response outcomes and correlations with tumoural GRPR expression from the phase I/IIa trial investigating the therapeutic use of [^177^Lu]NeoB in patients with [^68^Ga]NeoB avid disease (NCT03872778).

Finally, the authors wish to acknowledge the limitations of this study. SDH deficient wtGIST are rare and this is reflected in the sample size of this study. The patients included in this study were referred from multiple different centres and therefore the availability, type and timing of cross sectional imaging available for analysis was variable but helpful in excluding necrosis or haemorrhage as a cause for reduced or absent tracer uptake.

## Conclusion

[^68^Ga]NeoB PET imaging represents a potential adjunct in the management of wtGIST. While its heterogeneous uptake patterns and limited accuracy in detection of overall tumour burden limit its utility as tool for staging, the ability to target GRPR-positive lesions offers opportunities for personalized treatment strategies. Future research efforts should focus on elucidating the molecular determinants of GRPR expression and validating the clinical utility of [^68^Ga]NeoB PET imaging in guiding therapeutic interventions. An ongoing phase I/IIa trial is investigating the therapeutic use of [^177^Lu]NeoB in patients with [^68^Ga]NeoB avid disease, as identified via [^68^Ga]NeoB PET (NCT03872778).

## Supplementary Information

Below is the link to the electronic supplementary material.

Supplementary Material 1

## Data Availability

The datasets generated during and/or analysed during the current study are available from the corresponding author on reasonable request.

## Abbreviations

CT: Computed tomography
EBR: External beam radiotherapy
FDG: Fluorodeoxyglucose
GIST: Gastrointestinal stromal tumour
GRPR: Gastrin Releasing Peptide Receptor
MRI: Magnetic resonance imaging
PET: Positron emission tomography
PRRT: Peptide receptor radionuclide therapy
PSMA: Prostate specific membrane antigen
RFA: Radiofrequency ablation
SDH: Succinate dehydrogenase
SIRT: Selective Internal Radiation Therapy
SUV: Standardized uptake value
SUV_max_: Maximum standardized uptake value
SUV_mean_: Mean standardized uptake value
TKI: Tyrosine kinase inhibitor
Wt: Wild-type

## References

[1] Boikos SA, Pappo AS, Killian JK, LaQuaglia MP, Weldon CB, George S, et al. Molecular subtypes of KIT/PDGFRA Wild-Type Gastrointestinal stromal tumors: A report from the National institutes of health Gastrointestinal stromal tumor clinic. JAMA Oncol. 2016;2(7):922–8. 27011036 10.1001/jamaoncol.2016.0256 PMC5472100

[2] Janeway KA, Kim SY, Lodish M, Nose´ V, Rustin P, Gaal J, et al. Defects in succinate dehydrogenase in Gastrointestinal stromal tumors lacking KIT and PDGFRA mutations. Proc Natl Acad Sci USA. 2011;108:314–8. 21173220 10.1073/pnas.1009199108 PMC3017134

[3] Sosipatros A, Boikos Constantine A, Stratakis. The genetic landscape of Gastrointestinal stromal tumor lacking KIT and PDGFRA mutations. Endocrine. 2014;47:401–8. 25027296 10.1007/s12020-014-0346-3 PMC4729312

[4] van de Wal D, Elie M, Le Cesne A, Fumagalli E, den Hollander D, Jones RL, et al. Health-Related quality of life and side effects in Gastrointestinal stromal tumor (GIST) patients treated with tyrosine kinase inhibitors: A systematic review of the literature. Cancers. 2022;14:1832. 35406604 10.3390/cancers14071832 PMC8997462

[5] Van de Wiele C, Dumont F, Dierckx RA, Peers SH, Thornback JR, Slegers G, et al. Biodistribution and dosimetry of (99m)Tc-RP527, a gastrin-releasing peptide (GRP) agonist for the visualization of GRP receptor-expressing malignancies. J Nucl Med. 2001;42(11):1722–7. 11696645

[6] Waters MK, Cummings T-H, Jodrell H, et al. Increased gastrinreleasing peptide (GRP) receptor expression in tumour cells confers sensitivity to [Arg6,d-Trp7,9,NmePhe8]-substance P (6–11)-induced growth Inhibition. Br J Cancer. 2003;2(11):1808–16. 10.1038/sj.bjc.6600957 PMC2377129 12771999

[7] Carroll M, Chakrabarti, McDonald B. 1999. Aberrant expression of gastrin releasing peptide and its receptor by well-differentiated colon cancers in humans. Am. J. Physiol. Gastrointest. Liver Physiol. 1999; 276(3):655–665. 10.1152/ajpgi.1999.276.3.G655 10070042

[8] Scopinaro F, Varvarigou AD, Ussof W, De Vincentis G, Sourlingas TG, Evangelatos GP, et al. Technetium labeled bombesin-like peptide: preliminary report on breast cancer uptake in patients. Cancer Biother Radiopharm. 2002;17(3):327–35. 12136525 10.1089/10849780260179297

[9] Minamimoto R, Hancock S, Schneider B, Chin FT, Jamali M, Loening A, et al. Pilot comparison of 68Ga-RM2 PET and 68Ga-PSMA-11 PET in patients with biochemically recurrent prostate cancer. J Nucl Med. 2016;57(4):557–62. 26659347 10.2967/jnumed.115.168393

[10] Paulmichl A, Summer D, Manzl C, Rangger C, Orlandi F, Niedermoser S, et al. Targeting Gastrointestinal stromal tumor with 68Ga-Labeled peptides: an in vitro study on Gastrointestinal stromal tumor-Cell lines. Cancer Biother Radiopharm. 2016;31(8):302–31. 27754750 10.1089/cbr.2016.2092

[11] Dalm S, Bakker I, de Blois E, Doeswijk G, Konijnenberg M, Orlandi F, et al. 68Ga/177Lu-NeoBOMB1, a novel radiolabeled GRPR antagonist for theranostic use in oncology. J Nucl Med. 2017;58:293–9. 27609789 10.2967/jnumed.116.176636

[12] Virgolini I, Decristoforo C. Jun. MITIGATE study report. EudraCT number 2016-002053-38. 2019. https://www.clinicaltrialsregister.eu/ctr-search/trial/2016-002053-38/results

[13] Gruber L, Decristoforo C, Uprimny C, Hohenberger P, Schoenberg SO, Orlandi F, et al. Imaging properties and tumor targeting of ^68^Ga-NeoBOMB1, a Gastrin-Releasing peptide receptor antagonist, in GIST patients. Biomedicines. 2022;10(11):2899. 36428467 10.3390/biomedicines10112899 PMC9687401

[14] Djaileb L, Morgat C, Van de Veldt A, Virgolini I, Cortes F, Demange A, et al. Preliminary diagnostic performance of [68Ga]-NeoBOMB1 in patients with gastrinreleasing peptide receptor-positive breast, prostate, colorectal or lung tumors (NeoFIND). J Nucl Med. 2020;61(s1):346.

[15] Virgolini I, Wegener A, Cortes F. NeoFIND study report, 30 Mar 2020. Protocol number A005D-E01-201;EudraCT 2017-003432-37. https://clinicaltrials.gov/ct2/show/results/NCT03724253

[16] Dimitrakopoulou-Strauss A, Hohenberger P, Haberkorn U, Macke HR, Eisenhut M, Strauss LG. 68Ga-labeled Bombesin studies in patients with Gastrointestinal stromal tumors: comparison with 18F-FDG. J Nucl Med. 2007;48:1245–50. 17631559 10.2967/jnumed.106.038091

[17] Duan H, Iagaru A. PET imaging using Gallium-68 (^68^Ga) RM2. PET Clin. 2022;17(4):621–9. 36153233 10.1016/j.cpet.2022.07.006

[18] Stoykow C, Erbes T, Maecke HR, Bulla S, Bartholomä M, Mayer S, et al. Gastrin-releasing peptide receptor imaging in breast cancer using the receptor antagonist (68)Ga-RM2 and PET. Theranostics. 2016;19(10):1641–50. 10.7150/thno.14958 PMC4955063 27446498

[19] Ilias I, Chen CC, Carrasquillo JA, Whatley M, Ling A, Lazurova I, et al. Comparison of 6–18F-fluorodopamine PET with 123I-metaiodoben-zylguanidine and 111In-pentetreotide scintigraphy in localization of nonmetastatic and metastatic pheochromocytoma. J Nucl Med. 2008;49(10):1613–9. 18794260 10.2967/jnumed.108.052373 PMC2614907

[20] Bottoni G, Piccardo A, Fiz F, Siri G, Matteucci F, Rocca A, et al. Heterogeneity of bone metastases as an important prognostic factor in patients affected by oestrogen receptor-positive breast cancer. The role of combined [18F]Fluoroestradiol PET/CT and [18F]Fluorodeoxyglucose PET/CT. Eur J Radiol. 2021;141:109821. 34139575 10.1016/j.ejrad.2021.109821

[21] Hofman MS, Lau WF, Hicks RJ. Somatostatin receptor imaging with 68Ga DOTATATE PET/CT: clinical utility, normal patterns, pearls, and pitfalls in interpretation. Radiographics. 2015;35(2):500–16. 25763733 10.1148/rg.352140164

[22] van Berkel A, Rao JU, Kusters B, Demir T, Visser E, Mensenkamp AR, et al. Correlation between in vivo 18F-FDG PET and immunohistochemical markers of glucose uptake and metabolism in pheochromocytoma and paraganglioma. J Nucl Med. 2014;55(8):1253–9. 24925884 10.2967/jnumed.114.137034

[23] Berndsen M, Puls F, Thornell A, Arvidsson Y, Muth A, Lindskog S, Elias E. 2024. Gastrin-Releasing Peptide Receptor Expression in Gastrointestinal Stromal Tumours. ESMO *Gastrointestinal Oncology* 6 (December). 10.1016/j.esmogo.2024.100105 PMC12836489 41646945

[24] Emami B, Lyman J, Brown A, Coia L, Goitein M, Munzenrider JE et al. Tolerance of normal tissue to therapeutic irradiation. Int J Radiat Oncol Biol Phys. 991;21(1):109–22. 10.1016/0360-3016(91)90171-y 2032882

[25] Bresciani S, Garibaldi E, Cattari G, Maggio A, Di Dia A, Delmastro E, et al. Dose to organs at risk in the upper abdomen in patients treated with extended fields by helical tomotherapy: a dosimetric and clinical preliminary study. Radiat Oncol. 2013;8:247. 24160769 10.1186/1748-717X-8-247 PMC3816584

[26] Leonhard Gruber LD, Jiménez-Franco C, Decristoforo C, Uprimny G, Glatting P, Hohenberger SO, Schoenberg W, Reindl F, Orlandi M, Mariani W, Jaschke I, Virgolini. MITIGATE-NeoBOMB1, a phase i/iia study to evaluate safety, pharmacokinetics, and preliminary imaging of ^68^Ga-NeoBOMB1, a Gastrin-Releasing peptide receptor antagonist, in GIST patients. Journal Nuclear Medicine Dec. 2020;61(12):1749–55. 10.2967/jnumed.119.238808. 10.2967/jnumed.119.238808 32332143

[27] Ruigrok EAM, Verhoeven M, Konijnenberg MW, de Blois E, de Ridder CMA, Stuurman DC, Bertarione L, Rolfo K, de Jong M, Dalm SU. Safety of [^177^Lu]Lu-NeoB treatment: a preclinical study characterizing absorbed dose and acute, early, and late organ toxicity. Eur J Nucl Med Mol Imaging. 2022;49(13):4440–51. Epub 2022 Aug 11. PMID: 35951084; PMCID: PMC9605926. 35951084 10.1007/s00259-022-05926-2 PMC9605926

[28] Kurth J, Krause BJ, Schwarzenböck SM, Bergner C, Hakenberg OW, Heuschkel M. First-in-human dosimetry of gastrin-releasing peptide receptor antagonist [177Lu]Lu-RM2: a radiopharmaceutical for the treatment of metastatic castration-resistant prostate cancer. Eur J Nucl Med Mol Imaging. 2020;47(1):123–135. 10.1007/s00259-019-04504-3. Epub 2019 Sep 3. PMID: 31482426. 10.1007/s00259-019-04504-3 31482426

